# Low Anterior Resection Syndrome and Quality of Life of Patients After Sphincter Preservation Surgery: A Prospective Study

**DOI:** 10.7759/cureus.60059

**Published:** 2024-05-10

**Authors:** Chandramohan K, Akhil T Jacob, Madhu Muralee, Mira W Sudham, Mayadevi L, Sithara Balakrishnan

**Affiliations:** 1 Surgical Oncology, Regional Cancer Centre, Trivandrum, IND; 2 Nursing, Regional Cancer Centre, Trivandrum, IND

**Keywords:** diarrhea, post-operative complications, constipation, stoma, rectal cancer, functional evaluation, quality of life, low anterior resection syndrome

## Abstract

Background

After anterior resection (AR), one of the most debilitating complications is low anterior resection syndrome (LARS) seen in about 64% of patients. The severity of the LARS score was significantly correlated with neoadjuvant treatment, the extent of rectal surgery, complication by the anastomotic leak, female gender, and age < 64 years. In this study, we analyzed the impact of various clinical factors on LARS and also the various domains of quality of life (QoL).

Purpose

To assess the incidence of LARS in patients undergoing sphincter-sparing surgery for rectal cancer after the patient starts long-term defecating per anus, change in the QoL, and relation to LARS and factors affecting it.

Methods

One year before, 72 patients who had undergone AR in the Regional Cancer Centre were interviewed by a trained interviewer and data was collected from the file. The questionnaires used were the Wexner Incontinence score, LARS Malayalam Questionnaire, and European Organisation For Research and Treatment of Cancer (EORTC) QLQ C30 Malayalam translations.

Statistical measures

The LARS score was used to categorize patients into three grades. The scores were compared with clinical and social factors using the χ2 test for association. Continuous variables were compared by the Spearman Rho test.

Results

Details of patients were studied (male: 55.6% (40) and female: 44.4% (32)). Fifty patients underwent low anterior resection (LAR). The mean LARS score was 25.61, with 47.2% of patients having severe LARS score. The mean Wexner score was 6.84. The relation with type of surgery, approach (laparoscopic vs open), or type of neoadjuvant therapy was not found to be significant. A higher LARS score did not impact overall QoL as assessed by FACT-C. Insomnia and diarrhea symptoms scores were significantly worsened. The pain score was worse for those undergoing stapler anastomosis. Wexner's score was worse for those who had received adjuvant chemotherapy. Role functioning score was better for AR compared to low anterior resection (LAR). Only distance from the anal verge was found to be a significant cause of LARS and was negatively correlated.

Discussion

LARS of severe degrees were seen in most patients. No modifiable risk factors were significantly found to affect the chance of LARS. However, LARS did not have a significant impact on QoL, neither did the type of surgery. So sphincter preservation can be offered to the patients, but all patients undergoing LAR should be counseled well about the risk of LARS before surgery.

## Introduction

In the surgical management of rectal cancer, sphincter preservation is a much-desired outcome, provided oncological safety is not compromised. The surgical fraternity strives hard to perform low anterior resection (LAR) even for very low rectal cancers. When the oncologic outcome is equivalent, preserving the sphincter is thought to result in a better quality of life (QoL). However, this enthusiasm is tempered by the reality that up to 70% of patients may develop bowel dysfunction, which may affect their QoL, post-stoma reversal [1-3]. The dysfunction of the defecatory and continence mechanism after LAR is called low anterior resection syndrome (LARS). The complex symptoms of LARS include multiple or frequent defecations, the sensation of incomplete bowel emptying, repetitive attempts with the passage of small quantities of fecal matter within a few hours (clustering), fecal incontinence (which may range from mild fecal soiling or incontinence of flatus to significant incontinence of liquid or solid fecal matter), urgency (the disability of inability to retain fecal matter more than 15 minutes after the onset of the sensation to defaecate). In the initial Danish studies, LARS was seen in 64% of subjects, the severity being minor in about 23% of the patients and major LARS in 41% of the patients. [4,5] The cause of LARS is multifactorial, which includes muscular and neural causes [6]. The different contributors include weak puborectalis muscle, internal sphincter injury, or indirect neural damage due to neuropraxia of branches of the autonomic nervous system in the course of resection of the lesion or due to radiotherapy (RT), absence of the recto-anal inhibitory signals, and decreased rectal compliance and capacity after excision [6-11]. The management of LARS depends on its severity and major manifestations. For minor LARS, symptom-related management is warranted, while for severe LARS management includes sacral nerve stimulation and pelvic floor rehabilitation. This study was presented as a poster at the NATCON 2022 and was published as a preprint on 27/7/2023 at Research Square.

## Materials and methods

This was a prospective observational study of patients from January 2021 to January 2023. Seventy-two patients who had undergone sphincter-sparing surgery in the Regional Cancer Centre, followed by stoma reversal, one year prior to the date of the interview were interviewed by the trained interviewer, and data was collected. The following questionnaires were used to study the severity of AR syndrome: Wexner Incontinence score is a tool filled in by the interviewer. The questionnaire was developed by Dr. Steven Wexner from Cleveland Clinic [5]. This questionnaire incorporates five qualifying symptoms, which include flatus incontinence, liquid incontinence, solid incontinence, requirement to wear incontinence pads, and requirement for lifestyle alteration. Scoring includes adding up the individual scores, with a range of zero to 20, with a zero score representing the status of no incontinence, and a score of more than nine is designated as a clinically significant incontinence score. LARS Malayalam Questionnaire: This is a patient-administered questionnaire. The tool was developed by Emmertsen et al. [4]. It consists of five qualifying characteristics flatus incontinence, liquid stool incontinence, frequency, clustering of defecation, and urgency. Each symptom category is given individual weightage, and a cumulative score is obtained with a range from zero to 42. The severity of defecatory dysfunction was classified as absence of LARS (score of zero to 20), minor degree LARS (score of 21-29), and major degree LARS (score of 30-42). EORTC QLQ C30 Malayalam Translations: EORTC QLQ C30 is a colorectal malignancy-specific, patient self-administered questionnaire designed to determine QoL for patients with or cured of malignancy. The questionnaire comprises 30 questions, and these are further utilized to come to a global health or QoL index, with five functional domains (which include physical, emotional, functional role, social functioning, and cognitive), three symptom domain scales (pain, fatigue, and nausea or vomiting) and six single-item domain questions (diarrhea, constipation, insomnia, loss of appetite, dyspnea, and financial impairment). Scoring of the QoL is done as prescribed by the scoring manual as formulated in the EORTC QLQ-C30 questionnaire. The symptom and function scores undergo linear transformation to scales of zero to 100 points. An increased functional score represents a higher functionality (optimal score being 100). Contrarily an elevated symptom score represents an increase in the severity of symptoms (the optimal score being zero). This study used the Malayalam version of the score.

The questionnaire was administered once, one year after ileostomy reversal. All data was collected after clearance of the Human Ethics Committee, and after obtaining written informed consent. The study was conducted in accordance with the principles enshrined in the Declaration of Helsinki. Various factors affecting AR syndrome were analyzed with EORTC QoL, Wexner score, and LARS score. The factors like age and gender; disease-related factors like location and stage; and treatment-related factors like type of surgery, type of anastomosis, and use of chemoradiation were analyzed against QoL, LARS, and Wexner scores. The LARS was used to categorize patients into three grades. Wexner, LARS, and QoL scores were compared with demographic factors, tumor characteristics, and treatment options using a χ2 test for association or the Kruskal-Wallis test for nonparametric variables and the Mann-Whitney U test when appropriate. P-values <0.05 were considered statistically significant. Unifactorial ordinal logistic regression was used to identify risk factors related to impairment of scores.

## Results

Demographic details

The population comprised 40 males (55.6%) and 32 females (44.4%). Forty-three patients (59.7% of the population) were less than 60 years old. The mean age of the population is 56 with the range being from 28 to 75 years. Fifty-nine patients (92.2%) of the population had a good performance status of ECOG 0/1. The most common presenting complaint was bleeding per rectum, which was seen in 68 patients (83%), followed by constipation seen in 15 patients (20.8%). The mean distance from the anal verge was 7 cm with the distance ranging from 2 cm to 15 cm from the anal verge. Of the 72 patients, 40 (57.1%) had undergone neoadjuvant chemoradiotherapy (NACTRT) and 15 (21.4%) had undergone short-course radiotherapy (SCRT), with 17 receiving no form of neoadjuvant treatment. Thirty-four (47.2%) of the cases were done via open approach and 29 (40.3%) laparoscopically. Two cases were started as laparoscopic approaches and later converted to open. Sixty-eight patients (93%) had stapled anastomosis. All had temporary diversion ileostomy except three patients. Fifty patients (69.4%) had undergone low anterior resection (LAR), one (1.4%) had undergone supra levator exenteration (SLE), and one(1.4%) had undergone total proctocolectomy (TPC).

Functional scores

The mean LARS score was 25.61. Fifty-four patients (75%) had some form of LARS with 34 patients (47.2%) having severe LARS scores. The mean Wexner score was 6.84. The Chi-square analysis of factors, including gender, age, procedure done, approach of surgery, use of RT, and whether a partial mesorectal excision (PME) or total mesorectal excision (TME) was done, have been correlated with LARS (Table 1).

**Table 1:** Categorical factors associated with LARS score TME, total mesorectal excision; PME, partial mesorectal excision; AR, anterior resection; LARS, low anterior resection syndrome

Parameter		No LARS	Minor LARS	Major LARS	P-value
Gender	Male	9	9	22	0.32
	Female	9	11	12	
Age	<40	3	4	2	0.226
	41-50	2	3	8	
	51-60	5	6	10	
	61-70	3	7	10	
	>71	5	0	4	
Procedure	LAR	10	16	24	0.417
	AR	7	4	9	
	Supralevator exenteration	1	0	0	
	Total proctocolectomy	0	0	1	
Approach	Laparoscopic	10	8	11	0.525
	Open	8	11	17	
RT	Yes	16	18	21	0.637
	No	5	4	8	
TME vs PME	TME	12	9	24	0.742
	PME	1	2	4	

The relation of the type of surgery, approach (laparoscopic vs open), type of anastomosis (hand sewn vs stapled anastomosis), or type of neoadjuvant therapy were not found to be significantly related to LARS (Table 1).

When the correlation between LARS score and continuous variables was done, the time between neoadjuvant therapy to surgery, duration of temporary stoma, and age did not affect the scores. Rather only distance from the anal verge was found to be a significant cause of LARS. As the distance from the anal verge decreased, the chance of LARS increased (Table 2).

**Table 2:** Correlation of LARS scores with continuous variables (Spearman Rho test) *Significant correlation

Independent variables	Age	Distance from the anal verge	The time between neoadjuvant therapy to surgery	Duration of temporary stoma
Correlation coefficient	-0.063	-0.284*	-0.183	0.193
Sig. (two-tailed)	0.600	0.016	0.186	0.149

Quality of life indices

Duration for which the temporary stoma was maintained correlated with a poorer emotional component of FACT-C, even at the later point of time, that is, one year after the stoma was reversed. Further patients with higher age had more impairment in the emotional component of FACT-C. The relation of the QoL domains was compared to categorical and continuous variables and is shown in Tables 3, 4.

**Table 3:** Factors affecting quality of life (FACT-C) AR, anterior resection; TPC, total proctocolectomy; CTRT, chemoradiotherapy; SCRT, short-course radiotherapy; TME, total mesorectal excision; PME, partial mesorectal excision; QoL, quality of life; LAR, low anterior resection

Parameter		Total FACT-C ≤65.17	Total FACT-C 65.18-83.50	Total FACT-C >83.51+	P-value
Gender	Male	12	15	13	0.675
	Female	12	9	11	
Procedure	LAR	18	16	16	0.453
	AR	4	8	8	
	Supralevator exenteration	1	0	0	
	TPC	1	0	0	
Approach	Laparoscopic	12	7	10	0.625
	Open	11	14	11	
Neoadjuvant treatment	No	4	0	3	0.011
	CTRT	11	20	9	
	SCRT	5	0	4	
TME vs PME	TME	19	16	10	0.148
	PME	4	0	3	
Temporary stoma	No	2	1	3	0.294
	Yes	22	21	63	

**Table 4:** Correlation of FACT QoL score with continuous variables (Spearman Rho test) **Significant correlation coefficient LARS, low anterior resection syndrome; QoL, quality of life

QoL component (dependent variable)	Independent variables	Correlation coefficient	P-value
Social well-being	Age	0.163	0.171
	Distance from anal verge	-0.034	0.779
	Time between neoadjuvant therapy to surgery	-0.090	0.517
	Duration of temporary stoma	-0.066	0.625
	LARS	0.104	0.675
Emotional well-being	Age	.327**	0.005
	Distance from anal verge	-0.093	0.437
	Time between neoadjuvant therapy to surgery	0.122	0.380
	Duration of temporary stoma	.356**	0.007
	LARS	0.099	0.467
Functional well-being	Age	0.081	0.501
	Distance from anal verge	0.044	0.717
	Time between neoadjuvant therapy to surgery	0.170	0.220
	Duration of temporary stoma	0.220	0.101
	LARS	0.017	0.171
Additional concerns	Age	-0.041	0.730
	Distance from anal verge	-0.129	0.280
	Time between neoadjuvant therapy to surgery	-0.036	0.797
	Duration of temporary stoma	0.247	0.065
	LARS	-0.049	0.156
FACT-C total score	Age	0.161	0.177
	Distance from anal verge	-0.023	0.849
	Time between neoadjuvant therapy to surgery	0.092	0.510
	Duration of temporary stoma	0.237	0.076
	LARS	0.023	0.172
Physical functionality	Age	-0.137	0.251
	Distance from anal verge	-0.095	0.427
	Time between neoadjuvant therapy to surgery	0.025	0.858
	Duration of temporary stoma	-0.131	0.331
	LARS	-0.050	0.197
Role functioning cumulative score	Age	0.025	0.835
	Distance from anal verge	0.087	0.469
	Time between neoadjuvant therapy to surgery	-0.029	0.834
	Duration of temporary stoma	-0.065	0.630
	LARS	-0.016	0.182
Symptom scale: nausea and vomiting	Age	0.114	0.340
	Distance from anal verge	-0.029	0.811
	Time between neoadjuvant therapy to surgery	-0.209	0.129
	Duration of temporary stoma	0.114	0.399
	LARS	0.018	0.186
Emotional score	Age	.250*	0.034
	Distance from anal verge	0.101	0.397
	Time between neoadjuvant therapy to surgery	0.090	0.516
	Duration of temporary stoma	0.123	0.363
	LARS	-0.160	0.187
Cognitive functioning score	Age	0.029	0.812
	Distance from anal verge	0.121	0.311
	Time between neoadjuvant therapy to surgery	0.263	0.054
	Duration of temporary stoma	-0.037	0.786
	LARS	-0.116	0.563
Social functioning score	Age	-0.027	0.823
	Distance from anal verge	-0.031	0.799
	Time between neoadjuvant therapy to surgery	0.224	0.103
	Duration of temporary stoma	-0.239	0.073
	LARS	-0.169	0.112
Global QoL score	Age	0.033	0.785
	Distance from anal verge	0.125	0.297
	Time between neoadjuvant therapy to surgery	.346*	0.010
	Duration of temporary stoma	0.035	0.797
	LARS	0.138	0.221
Dyspnea score	Age	-0.025	0.834
	Distance from anal verge	0.130	0.277
	Time between neoadjuvant therapy to surgery	-0.111	0.426
	Duration of temporary stoma	-0.216	0.106
	LARS	-0.275	0.223
Insomnia score	Age	-0.112	0.350
	Distance from anal verge	-0.147	0.218
	Time between neoadjuvant therapy to surgery	-0.234	0.089
	Duration of temporary stoma	0.176	0.192
	LARS	0.240	0.042
Loss of appetite score	Age	-0.185	0.119
	Distance from anal verge	0.082	0.493
	Time between neoadjuvant therapy to surgery	-0.193	0.161
	Duration of temporary stoma	-0.128	0.344
	LARS	0.085	0.112
Nausea and vomiting score	Age	-0.116	0.331
	Distance from anal verge	-0.011	0.929
	Time between neoadjuvant therapy to surgery	-0.098	0.481
	Duration of temporary stoma	-0.234	0.080
	LARS	0.018	0.221
Constipation score	Age	-0.006	0.959
	Distance from anal verge	-0.075	0.529
	Time between neoadjuvant therapy to surgery	-0.017	0.904
	Duration of temporary stoma	0.077	0.568
	LARS	-0.096	0.211
Diarrhea score	Age	-0.076	0.525
	Distance from anal verge	-0.128	0.283
	Time between neoadjuvant therapy to surgery	0.012	0.933
	Duration of temporary stoma	-0.111	0.412
	LARS	0.254	0.031
Pain score	Age	-0.066	0.583
	Distance from anal verge	0.015	0.901
	Time between neoadjuvant therapy to surgery	-0.192	0.163
	Duration of temporary stoma	0.076	0.576
	LARS	0.044	0.112
Financial difficulty score	Age	0.044	0.713
	Distance from anal verge	0.059	0.626
	Time between neoadjuvant therapy to surgery	-0.179	0.195
	Duration of temporary stoma	0.165	0.219
	LARS	0.138	0.986

Patients who had undergone some forms of neoadjuvant therapy were found to have a worse QoL score. However, other modifiable factors were less likely to impact overall QoL.

A higher LARS score did not impact overall QoL as assessed by FACT-C as well as the global QoL as determined by the EORTC C30 score. However, the patients performed poorer in the diarrhea and insomnia symptom domains. With worse LARS scores, patients had a trend toward worse functional outcomes; however, this was not found to be statistically significant. None of the patients had received any treatment directed at LARS.

## Discussion

In our population, the prevalence of LARS was 75% (54). Major LARS was seen in 47.2% (34) of the patients, which is similar to that found in most of the published data [4,8,12]. A study done in Italy reported long results indicating the prevalence and severity of the LARS score at a point more than 10 years after resection (93 participants were included with the median follow-up being 13.7 years) [13]. The study by Chen et al, done in 242 patients, with a median follow-up of 14.6 years showed that the LARS prevalence was 68%, which was of minor degree in 22% and of major severity in 46% [13]. No modifiable risk factors were significantly found to affect the chance of LARS. The only factor that was found to have a statistically significant relation to LARS was the distance from the anal verge. This may be due to the need for more dissection and difficulties encountered during low resection. However, the type of surgery was found not to be statistically significant in its relationship with LARS. This may be an indicator that the damage to surrounding structures and surgical difficulty may be more important than the level of anastomosis. According to the data from the Danish study group, the degree of LARS had a significant correlation with neoadjuvant therapy (with relative risk (RR) of 2.48), the extent of rectal surgery TME vs PME (with RR of 2.31), complication in the form of anastomotic leakage (RR being 2.06), younger chronological age (age less than 64 years old) (RR: 1.90), and female gender (RR: 1.35) [14,15]. The severity of the LARS score was maximum if the patient had undergone NACTRT + TME, compared to PME or TME alone. It usually takes approximately one year after surgery for the symptoms to plateau after which there will not be significant improvement. From the same data, Wiltink et al. noted that RT had a statistically significant impact on LARS when they compared the outcomes of the 241 patients who had undergone RT and those who did not do so (237 patients) with the median follow-up being 14 years [12].

The reassuring aspect that has been determined in this study is that LARS did not have a significant impact on QoL. However, in comparison to the data from the same institution, the mean QoL score as determined by FACT-C was better for patients post-APR (74.35) when compared to patients with LARS (69.5). This may be due to the social limitations, wherein the public lavatory facilities may not be available readily, and the manual nature of the occupation of patients significantly impairing the functionality of the patients with LARS. Furthermore, on assessment of the symptom domains, patients with LARS had worse diarrhea scores and worse insomnia scores. The incomplete evacuation has resulted in increased frequency, which is perceived as diarrhea, and it is significant enough to affect sleep. Thus, patients were significantly affected by the LARS, with respect to bowel function and sleep; however, the effect of this may have been neutralized by the lack of significant difference in the social well-being and emotional score, which may be due to a permeating desire to avoid stoma, better family support, social accommodation, and robust stoma care and functional counseling provided, which prepares the patients to better tolerate the compromises of the LARS, thus protecting the overall QoL. So, sphincter preservation can be offered to the patients, explaining the possibility of developing LARS with proper counseling of the patient and their family members, so as to tide over the functional impairment.

The elderly population and those who have temporary stomas retained for longer periods of time are more likely to have worse emotional scores, as assessed by FACT-C. Prolonged retention of stoma, especially with the fluid nature of the ileal effluent, may significantly hamper the emotional status of the patients, which leaves behind a trauma that has a lasting impact on the patient’s emotional well-being even as late as one year. This may act as an incentive toward considering early stoma reversal as much as feasible so as to improve the QoL and spare the patients from lasting emotional trauma. The elderly population is prone to emotional lability and depression due to age, comorbid issues, and sociocultural deficiencies. So the LARS can act as an additional stressor, which hampers the emotional QoL for this population. This is a small study involving 72 patients and hence there are limitations to the extent to which conclusions can be drawn from this. Furthermore, the assessment reported herein is at a single point in time so the dynamic changes have not been measured in this report, the assessment of which is still underway. In previous studies, it has been seen that the QoL outcomes reach a nadir by six months and then gradually improves [16].

## Conclusions

In a society that very much desires a stoma-free life, sphincter-preserving procedures if oncologically safe can be offered. However, the patients have to be educated that their symptomatology may be significantly disturbing, and they may have a better QoL with a permanent stoma. The patients should be counseled appropriately, regarding what to expect and the family should be able to support the patient. The societal mechanisms should evolve to better help and support these individuals by providing sanitary facilities and public lavatories. This study is limited by the small sample size and further studies into this matter would be required to further elucidate the details and real-world impact of LARS.
